# Analysis of chikungunya virus proteins reveals that non-structural proteins nsP2 and nsP3 exhibit RNA interference (RNAi) suppressor activity

**DOI:** 10.1038/srep38065

**Published:** 2016-11-30

**Authors:** Kalika Mathur, Abhishek Anand, Sunil Kumar Dubey, Neeti Sanan-Mishra, Raj K. Bhatnagar, Sujatha Sunil

**Affiliations:** 1 Insect Resistance Group, International Centre for Genetic Engineering and Biotechnology, New Delhi, India; 2 Plant Molecular Biology Group, International Centre for Genetic Engineering and Biotechnology, New Delhi, India

## Abstract

RNAi pathway is an antiviral defence mechanism employed by insects that result in degradation of viral RNA thereby curbing infection. Several viruses including flaviviruses encode viral suppressors of RNAi (VSRs) to counteract the antiviral RNAi pathway. Till date, no VSR has been reported in alphaviruses. The present study was undertaken to evaluate chikungunya virus (CHIKV) proteins for RNAi suppressor activity. We systematically analyzed all nine CHIKV proteins for RNAi suppressor activity using Sf21 RNAi sensor cell line based assay. Two non-structural proteins, namely, nsP2 and nsP3 were found to exhibit RNAi suppressor activity. We further validated the findings in natural hosts, namely in *Aedes* and in mammalian cell lines and further through EMSA and *Agrobacterium* infiltration in GFP silenced transgenic tobacco plants. Domains responsible for maximum RNAi suppressor activity were also identified within these proteins. RNA binding motifs in these domains were identified and their participation in RNAi suppression evaluated using site directed mutagenesis. Sequence alignment of these motifs across all species of known alphaviruses revealed conservation of these motifs emphasizing on a similar role of action in other species of alphaviruses as well. Further validation of RNAi suppressor activity of these proteins awaits establishment of specific virus infection models.

RNA interference (RNAi) is a conserved evolutionary mechanism designed to impart an antiviral defence system across organisms ranging from plants, fungi, arthropods and nematodes^1^,^2^,^3^,^4^. The phenomenon, triggered by the production of double stranded RNA (dsRNA) by the pathogen upon infection, leads to the activation of the RNA interference silencing complex (RISC) by loading of dsRNA/siRNA into the complex ultimately leading to cleavage of the viral RNA^5^,^6^,^7^. Several components of RISC participate in the cleavage, most important being ribonuclease III Dicer (DCR) and Argonaute protein (AGO). Dicer acts on dsRNA to generate small RNA (sRNA) that are loaded into the AGO, resulting in cleavage^8^,^9^; however, some RNAi pathways are independent of Dicer and other components like RNA-dependent RNA polymerase (RdRP) produce short RNA transcripts that directly bind to AGO^10^. Similarly, much is now known about the Piwi pathway that is specifically active in the germline^11^.

In response to RNAi in plants and insects, viruses infecting these systems utilise some of their own proteins/sequence elements that counteract the antiviral RNAi pathway^12^. These viral suppressors of RNAi (VSRs) act through different mechanisms, either by binding to long dsRNA and protecting them from Dicer^7^,^13^ or short RNAs^14^ or through multi-mode mechanisms^15^,^16^. Recently it has been shown that some viruses encode host-specific VSRs due to co-evolution of the virus and the host, emphasising on the importance of analysing suppressor activity in relevant host^17^.

Amongst medically important pathogenic viruses, arboviruses form an important subset of viruses due to the complexity of their host preferences^18^. These viruses, mainly constituted by genera flavivirus and alphavirus, are transmitted through arthropods, and display differential replication pattern in the two hosts. While invertebrate hosts like mosquitoes and ticks behave as maintenance hosts where the viruses persists in low levels^19^,^20^, vertebrate hosts get infected to high titers with efficient virus replication^21^ and this alteration between hosts has been shown to play an important role in evolution of arboviruses^22^. Until recently, it had been speculated that these arbovirus do not possess RNAi suppressor activity^23^ unlike other insect-specific viruses like FHV and CrPV^24^,^25^. However, recent studies have established that flaviviruses encode for VSRs^26^,^27^. Alphaviruses are member of arboviruses containing 27 recognised members^28^ with a very wide geographic distribution and several geographical variants on the basis of which they have been classified as Old World and New World viruses^29^. Grouped into seven complexes based on serological cross-reactivity, they infect a variety of host including birds, fishes, mammals including humans and are maintained in natural cycles by transmission between susceptible vectors and vertebrate hosts^30^. Alphaviruses have a single plus- stranded RNA genome encapsulated by capsid proteins. The approx. 12 kb genome consists of two open reading frames that encodes four non-structural proteins (nsP1 to nsP4), three structural proteins (capsid, envelope glycoproteins E1 and E2) and two small cleavage products (E3 and 6K). While the structural proteins are involved in mediating entry and fusion of the virus into the hosts, the non-structural proteins (nsPs) form the replication complex^31^. The non-structural proteins that are initially translated as one or two polyprotein from the full-length genomic viral RNA are processed exclusively by the virus-encoded protease, nsP2. Upon cleavage, each of the mature proteins as well as the intermediates actively participates in virus replication. As of date, some properties of these nsPs are known^32^: nsP1 is membrane-associated and possess both guanine-7-methytransferase and guanyltransferase activity, nsP2 exhibit several enzymatic activities including helicase, protease activity, nsP3 possess phosphatase and RNA-binding activity^33^,^34^ and nsP4 is a RNA-dependent RNA polymerase (RdRP)^35^. Even though much information is available from the above and other studies, it is expected that these proteins have more novel roles in viral replication.

The current study undertaken to identify viral encoded suppressors of RNAi in chikungunya virus (CHIKV) has thrown light to possible RNAi suppressor activity in two non-structural proteins of CHIKV. Systematic analysis of all CHIKV proteins using a Sf21 RNAi sensor cell line based assay revealed that non-structural proteins nsP2 and nsP3 exhibited RNAi suppressor activity. We further validated this finding in its natural hosts, namely, an *Aedes* and mammalian cell line. After dissecting the domain responsible for suppressor activity, we evaluated the suppressor strength by mutating the RNA binding motifs in these domains. Aligning known sequences of nsP2 and nsP3 across all alphavirus species, we observed that these RNA binding motifs are conserved across species thereby confirming the importance of these motifs in alphavirus-host interactions vis-à-vis RNAi pathway.

## Results

### Identification of viral suppressors of RNAi (VSRs) in chikungunya virus (CHIKV)

**Figure 1:**
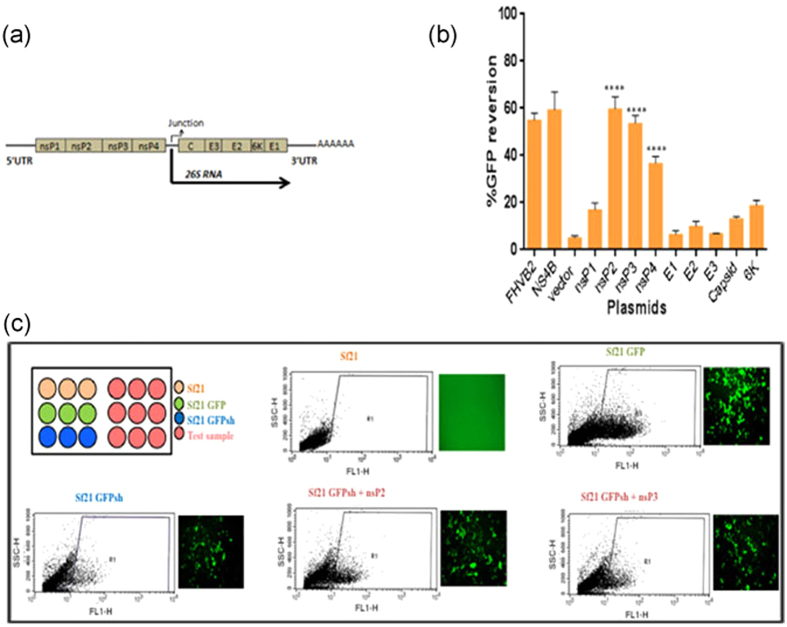
Screening of CHIKV genes for RNAi suppressor activity. (**a**) Schematic representation of CHIKV genome, showing non-structural and structural genes. (**b**) GFP reversion assay of all CHIKV genes in Sf21 sensor cell line. FHVB2 and NS4B were used as positive controls. The y-axis shows % GFP reversion (Mean ± SD). Statistical significance was analysed using student’s t-test and Fisher’s Least Significant Difference (LSD) test using empty vector as control. The *symbol indicates a statistically significant difference in terms of the P value (P < 0.001). nsP2, nsP3 and nsP4 were found statistically highly significant from LSD test (P value < 0.0001 each) when compared with the vector (negative control) (Alpha level of the test = 0.05). (**c**) Schematic representation of transfection methodology for Sf21 sensor cell line along with fluorescent microscopic images and FACS dot plot 72 h post transfection. Sf21 and Sf21 GFP stable cell lines were used as FACS gating control. All the transfections were performed in Sf21 sensor cell line having GFPshRNA stably integrated.

Studies using recombinant alphavirus with an endogenous RNAi suppressor have shown an increase in alphavirus replication in *Aedes* mosquitoes^36^. While it was previously thought that flaviviruses do not possess any RNAi suppressors, it was recently proved that these viruses do possess VSRs that are active in both insect and mammalian cells^26^,^27^. In case of alphaviruses, reports have suggested that RNAi suppressors in this class of viruses may either be weak or undetected due to inappropriate assay conditions^4^,^37^. We undertook the present study to systematically evaluate CHIKV proteins (Fig. 1a) for possible RNAi suppressor activity using a Sf21 RNAi sensor cell line developed in our lab^16^. Using this RNAi sensor cell line, in which GFPshRNA is expressed constitutively to silence GFP in a GFP Sf21 cell line; we checked for reversion of GFP due to RNAi suppressor activity of the cloned CHIKV genes in pIB vector using flow cytometry (Fig. 1b and c). Post 72 h transfection, it was observed that nsP2, nsP3 and nsP4 showed a reversion in GFP expression of 60%, 55% and 40% respectively (p values < 0.0001). The other genes showed reversion similar to empty vector. DENV RNAi suppressor NS4B and flock house virus protein B2 served as positive controls.

### CHIKV proteins nsP2 and nsP3 exhibit RNAi suppressor activity in natural host cell lines

**Figure 2:**
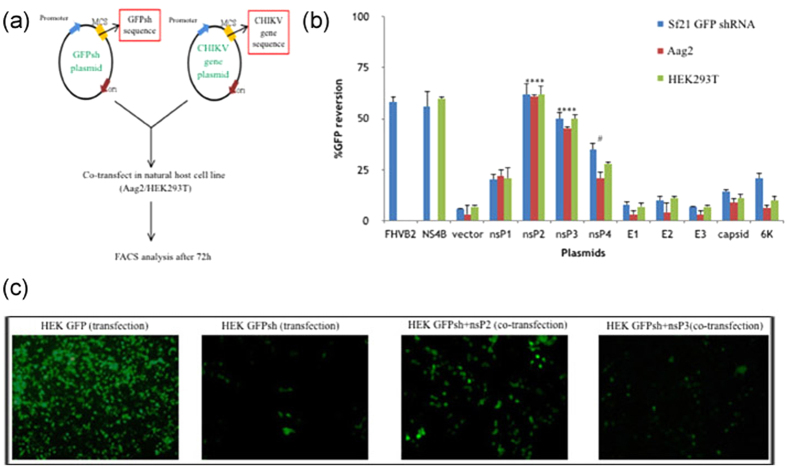
Validation of RNAi suppressor activity in natural host cell lines. (**a**) Workflow of transient transfections in natural host cell lines (Aag2 and HEK293T). The cells were co-transfected with host specific GFPshRNA plasmid and CHIKV gene plasmid. GFP based FACS analysis was done after 72 h. (**b**) Graphical representation of % GFP reversion (Mean ± SD) in different cell lines, namely Sf21 GFPshRNA, Aag2 and HEK293T. Previously published VSRs FHVB2 (flock house virus) and NS4B (dengue virus) were taken as positive controls and empty vector was used as negative control. Statistical significance was analysed using student’s t-test and Fisher’s Least Significant Difference (LSD) test using empty vector as control. The *symbol indicates a statistically significant difference in terms of the P value (P < 0.001) and ^#^symbol represents p-value > 0.001. nsP2 (p-value = 0.0003) and nsP3 (p-value = 0.0007) were found statistically highly significant, while nsP4 was found not significant (p-value = 0.035) (Alpha level of the test = 0.05). (**c**) Fluorescent microscopic images of HEK293T cell line based assay. HEK293T cells were transfected with GFP plasmid and GFPshRNA plasmid in separate wells. For GFP reversion assay GFPshRNA and individual VSR plasmid were co-transfected and cells were checked for fluorescence under microscope at 72 h post transfection.

Previous studies have emphasised the importance of testing the efficiency of VSRs in relevant host systems^17^. Having checked suppressor activity of all CHIKV genes using an established cell line based assay, we next checked for their suppressor activity in its natural hosts, namely a mosquito and mammalian system. For this purpose, we utilised an *Aedes* cell line, Aag2 and the mammalian cell line, HEK293T. Using transient transfection protocols in which GFPshRNA in pIZT/V5-His and pGFP VRS plasmid were co-transfected with nsP2/nsP3 pIB/pCDNA3.1+ plasmids in Aag2 and HEK293T respectively (Fig. 2a), we evaluated suppressor activity of all nine genes. Overall, it was seen that transfection worked better in HEK as compared to Aag2 owing to better transfection efficiency in the mammalian system. With respect to GFP reversions, it was observed that nsP2 and nsP3 showed significant reversion (p value < 0.001) in both *Aedes* and HEK cell lines (Fig. 2b and c). In case of nsP4, however, RNAi suppressor activity was not evident in both the natural hosts and only around 25% GFP reversions were seen in the cells upon transfections with nsP4. Based on these results, we identified CHIKV_nsP2 and CHIKV_nsP3 to possess RNAi suppressor activity and further proceeded to validate these findings.

### nsP2 and nsP3 are suppressors of RNAi

**Figure 3:**
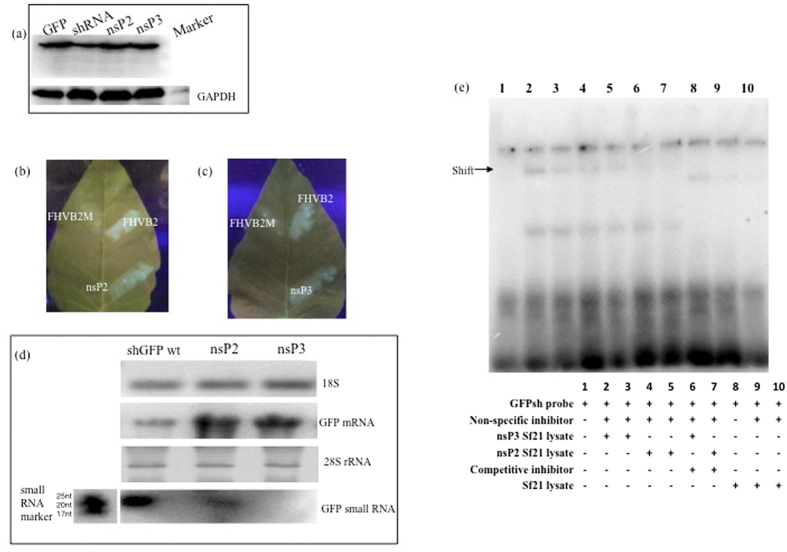
*In vitro* and *in vivo* assays to validate VSR activity. (**a**) Western blotting to show changes in GFP levels upon transfection with nsP2 and nsP3. Sf21 sensor cell line was transfected with VSRs and western blotting was done using anti-GFP antibody. GADPH was used as housekeeping control. (**b** and **c**) CHIKV nsP2 and nsP3 show RNAi suppressor activity in *in vivo* system. Transgenic *Nicotiana* leaves with GFPshRNA stably integrated were infiltrated with VSR expressing *Agrobacterium* cultures and checked for GFP reversion under UV transilluminator. FHVB2 was used as positive control and mutated FHVB2 was the negative control. FHVB2M shows necrosis marks due to infiltration, but no GFP reversion was seen. (**d**) Northern blotting to show changes in GFP mRNA and small RNA levels upon VSR infiltration in *Nicotiana* leaves. RNA isolated from infiltrated leaves was used to detect GFP mRNA levels using GFPshRNA oligonucleotide end labelled with [γ32P] ATP. 18 S was used as housekeeping control. GFP small RNA population was detected by northern blotting using 700 bp DIG labelled GFP probe. 28SrRNA was used as house keeping control. (**e**)Electrophoretic mobility shift assay (EMSA) using labeled GFPshRNA probe and VSR transfected Sf21 cell lysate. GFPshRNA oligonucleotide was end-labelled with [γ32P] ATP and mixed with different concentrations of VSR transfected Sf21 cell lysate. Lane 1: Free shRNA probe; lane 2, 3, 4, 5: Different concentrations of nsP3 (30 μg, 20 μg) and nsP2 (30 μg, 20 μg) transfected Sf21 lysate respectively; lane 6&7: nsP3 and nsP2 transfected Sf21 cell lysate with 100 fold unlabelled GFPshRNA probe; lane 8, 9 & 10: binding of untransfected Sf21 cells to GFPshRNA in the absence and presence (1 μg & 2 μg) of uncompetitive inhibitor. Salmon sperm DNA (2 μg) was used as non specific inhibitor in all binding reactions.

Having established that nsP2 and nsP3 show RNAi suppressor activity, we proceeded to detect the level of GFP protein due to effect of the putative VSRs. Western analysis also revealed increase in GFP expression in nsP2 and nsP3 transfected cells as compared to Sf21 RNAi sensor cells (Fig. 3a). In addition to the above mentioned *in vitro* assays, we utilised transgenic *Nicotiana* leaf based assay to validate RNAi suppressor activity of nsP2 and nsP3 under *in vivo* conditions^38^. The putative VSRs were cloned into the plant binary vector pBI 121 and reversal of silencing by agro-infiltration-mediated transient ectopic expression of CHIKV nsP2 and CHIKV nsP3 was assessed in transgenic tobacco leaf tissues in which GFP is silenced. The infiltrated leaves showed reversal of GFP activity at 3 days post-infiltration in nsP2 and 5 days post-infiltration in nsP3 as seen under UV light (Fig. 3b and c). In the infiltration experiments, FHVB2 plasmid was used as positive control and a mutated FHVB2 expressing plasmid as negative control. GFP reversal was further confirmed by analysing GFP transcript levels and the depletion of small RNA population of GFP in the leaves using Northern hybridisation. 18 s RNA and 28 s rRNA were used as loading controls for mRNA and small RNA Northerns respectively. (Fig. 3d). While there was an increase in the GFP mRNA levels in leaves infiltrated with nsP2 and nsP3, GFP small RNAs showed 50% and 80% decrease in their population in the leaves infiltrated by nsP2 and nsP3 respectively thereby providing evidence that CHIKV nsP2 and nsP3 are influencing small RNA production post-transcription.

Even as the assays in different systems provided enough clarity as to presence of RNAi suppressor activity in CHIKV nsP2 and nsP3, the first strong proof for any RNAi suppressors is its role in interfering with the RNAi machinery. The most well known mechanism is the ability of VSRs to bind to dsRNA/siRNA population thereby preventing RISC from acting upon them. This phenomenon has been evaluated by studying the binding of suppressor protein to double stranded RNA population resulting in a shift in their mobility efficiency^7^,^16^. For this purpose, we expressed both nsP2 and nsP3 in the heterologous *E.coli* system. While we were able to produce nsP3 in its natural folded form, nsP2 aggregated into inclusion bodies and could not be purified, most probably owing to its large size. To overcome this problem, we expressed both CHIKV_nsP2 and CHIKV_nsP3 in Sf21 cell line by transient transfection (Supplementary Fig. S2). Using cell lysate of the transfected cell expressing nsP2 and nsP3, we evaluated their ability to bind double stranded RNA by using [γ32P] labeled shRNA of GFP and running the bound complex in a 6% polyacrylamide gel (Fig. 3e). Binding of shRNA to nsP2 and nsP3 proteins were evident by complex formation and shift that increased proportionally to protein concentration (Fig. 3e-lane no. 2, 3, 4, 5). Addition of cold shRNA probe served as competitive inhibitor of binding reaction and the decrease in the intensity of the shift proved the binding specificity of the complex. Untransfected Sf21 lysate in the presence of non-specific inhibitor (salmon sperm DNA) of varying concentrations and GFPshRNA served as negative controls (Fig. 3e-lane 1, 8, 9, 10). These results clearly proved nsP2 and nsP3 bound to dsRNA. Taken all these findings together, we conclude that CHIKV nsP2 and CHIKV nsP3 are suppressors of RNAi.

### CHIKV RNAi suppressors function downstream of RISC loading in the RNAi pathway

**Figure 4:**
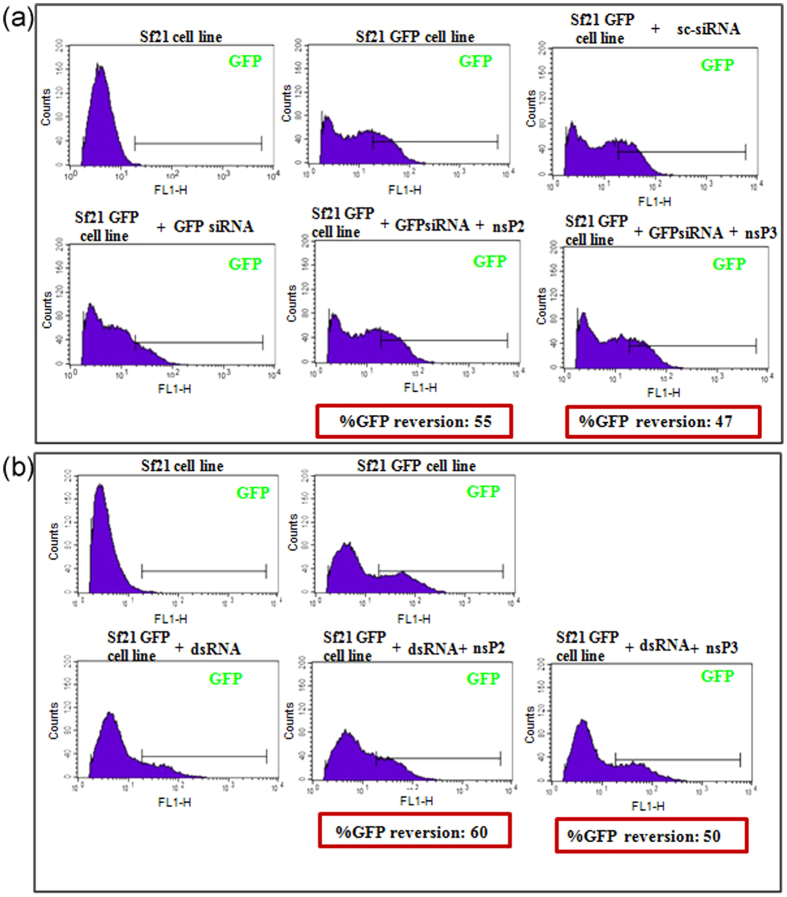
Reversion assay of GFP dsRNA and siRNA mediated silencing. (**a**) Sf21 GFP stable cell line was silenced with 2.5 pmole GFP siRNA. Scrambled siRNA (sc-siRNA) was used as siRNA negative control. GFP reversion assay was performed by co-transfecting GFP siRNA and VSR plasmids. FACS analysis was done after 72 h. (**b**) Sf21-GFP stable cell line was silenced by transient transfection with GFPdsRNA plasmid. Reversion assay was performed by co-transfecting GFPdsRNA plasmid and VSR plasmid. FACS analysis was done after 72 h. From the FACS data percentage GFP reversion was calculated as ((percentage GFP upon co-transfection) − (percentage GFP after silencing)/(percentage GFP of GFP cell line) − (percentage GFP after silencing)) × 100.

Virus suppressor proteins interfere with the host RNAi components to inhibit the pathway. VSRs may block the dicing activity and thereby reduce formation of siRNAs. Alternately, VSRs may also impede loading of siRNAs into the RISC and consequently block functionalization of siRNA loaded RISC^37^.A direct way to assess effect of VSRs on siRNA formation is to estimate dicing enzyme activity *in vitro* in the presence of the VSRs. Unfortunately, our attempts to purify CHIKV nsP2 in its active form were unsuccessful, hence we resorted to cell based FACS assays to gain preliminary insight into possible suppression mechanism. Using SF21 GFP cell line, we transfected dsRNA or siRNA- GFP and subsequently co-transfected with the VSRs to estimate the level of GFP reversal. We reasoned that GFP reversal was an indication of where the VSRs may act – if they acted during dicing, then the reversal would be evident upon co-transfection with dsRNA+VSRs and if they acted at the time of siRNA loading into RISC, it would be evident while using siRNA+VSRs. Our results showed there was >50% GFP reversion with both dsRNA and siRNA for both nsP2 and nsP3 (Fig. 4a and b), thus highlighting that their mode of action may be downstream of RISC loading in the RNAi pathway.

### Domain mapping of nsP2 and nsP3 reveal domain specific RNAi suppressor activity

**Figure 5:**
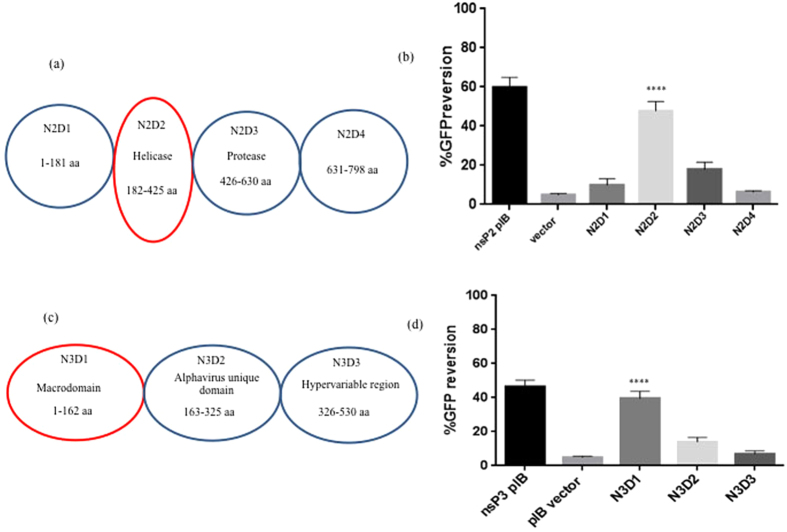
Domain mapping of VSR activity. (**a** and **c**) Schematic representation of different domains of nsP2 (N2D1, N2D2, N2D3 and N2D4) and nsP3 (N3D1, N3D2 and N3D3).(**b** and **d**) Graphical representation of Mean ± SD of FACS data for GFP reversion in individual domains of VSRs. The domains were separately cloned in pIB/V5-His TOPO vector and transfected in Sf21 sensor cell line. GFP based FACS analysis was done after 72 h. Percentage GFP reversion was calculated from FACS data. Statistical significance was analysed using student’s t-test and Fisher’s Least Significant Difference (LSD) test using empty vector as control. The *symbol indicates a statistically significant difference in terms of the P value (P < 0.001). nsP2 domain2 (N2D2 p-value < 0.0001) and nsP3 domain 1 (N3D1 p-value < 0.0001) were found statistically highly significant (Alpha level of the test = 0.05).

The non-structural proteins, nsP2 and nsP3 of CHIKV participate in several functions; nsP2 has been shown to have multiple functions including helicase, protease and NTPase activities^39^,^40^,^41^,^42^. This protein is 90 kDa and consists of four domains, the 180 aa long N-terminal domain, 240 aa long domain that exhibits helicase activity, 200 aa long protease domain and a 160 aa long C terminal domain^43^ (Fig. 5a). Similarly, nsP3 exhibits phosphatase activity and also consists of a macrodomain that has been well characterised^32^. This is 57 kDa and consists of three domains (Fig. 5c). The macrodomain, present on the N-terminus, is a globular domain 162 aa long and is evolutionary highly conserved. Most of the activities exhibited by the protein are associated with this domain. The sequence of central part of nsP3 (162 aa) is conserved only among alphaviruses and hence is termed alphavirus unique domain (AUD). The C-terminal domain (204 aa) is termed hypervariable region, since it has no sequence similarity even among alphaviruses. Having confirmed that both nsP2 and nsP3 exhibit RNAi suppressor activities, efforts were taken to characterise the proteins better and to identify those specific domains that were responsible for RNAi suppressor activity. For this purpose, each of the domains was cloned individually in pIB/V5-His TOPO vector and transfected into the Sf21 sensor line. For convenience, the domains were labelled as N2D1, N2D2, N2D3 and N2D4 in nsP2 and N3D1, N3D2 and N3D3 in nsP3. In case of nsP2, N2D2, the domain that is responsible for helicase activity showed maximum reversion of GFP expression and in nsP3, N3D1, i.e., the macrodomain showed maximum reversion (Fig. 5b and d).

### RNA binding motifs are conserved across alphaviruses

**Figure 6:**
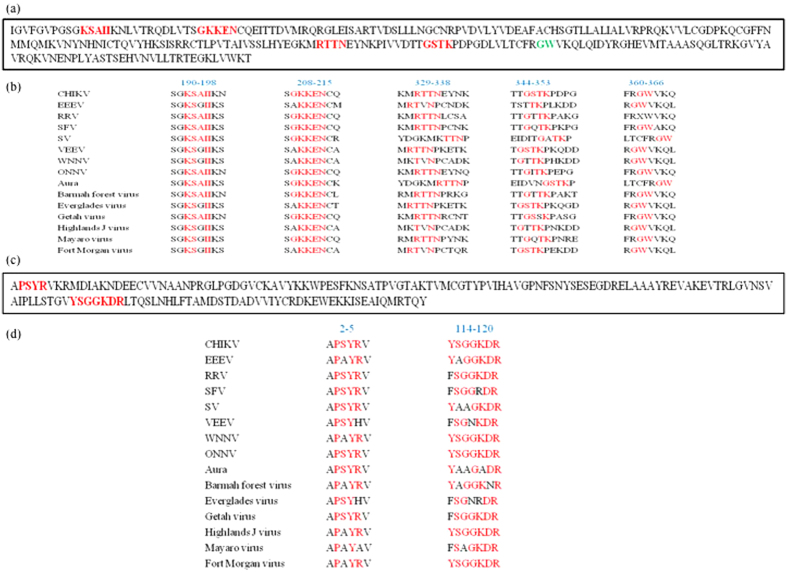
Conservation of RNA binding motifs of VSRs across alphavirus species. (**a** and **c**) BindN predicted RNA binding motifs in CHIKV nsP2 domain 2(N2D2) and nsP3 domain 1(N3D1). (**b** and **d**) Alignment of alphaviruses to check for conservation of putative RNA binding motifs.

It is well known that most of the VSRs possess certain sequence characteristics to function as an RNAi suppressor. Presence of RNA binding motifs and presence of GW/WG motifs has been shown to be important features required for binding to dsRNA/siRNA and Argonaute loading^44^. To understand the sequence structure of the domains that showed maximum suppressor activity in nsP2 and nsP3, we identified the RNA binding motifs within the domains showing maximum reversions in both the VSRs (Fig. 6a and c). Furthermore, we hypothesized that these features are important amongst all species of alphavirus. To test this hypothesis, we aligned the sequences of all known alphaviruses nsP2 and nsP3. To date, complete sequence information is available for 15 alphavirus species. As expected, all RNA binding motifs were conserved in both nsP2 and nsP3 proteins across all species (Fig. 6b and d). Additionally, we observed the presence of one GW motif in domain 2 of nsP2 in all species. Taking all these aspects, we hypothesize the RNAi suppressor activity of CHIKV nsP2 and nsP3 may be conserved across all alphaviruses.

### RNA binding motifs contribute to VSR activity of CHIKV nsP2 and nsP3

**Figure 7:**
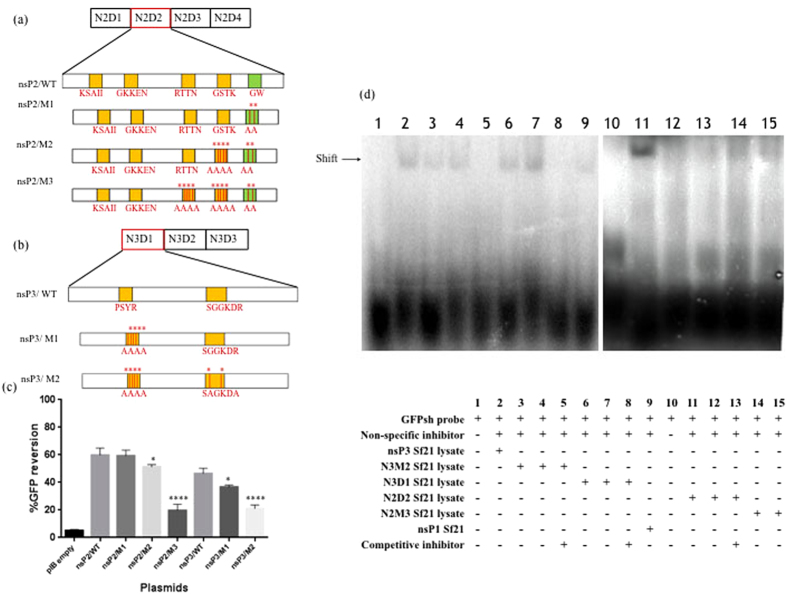
Effect of RNA binding motifs on RNAi suppressor activity. (**a** and **b**) Schematic representation of site directed mutagenesis at multiple sites in nsP2 and nsP3 pIB/V5-His TOPO plasmids where nsP2/M1, nsP2/M2 and nsP2/M3 refer to different motifs mutated in nsP2 pIB/V5-His TOPO plasmid; while nsP3/M1and nsP3/M2 refer to different motifs mutated in nsP3 pIB/V5-His TOPO plasmid. RNA binding motifs predicted in N2D2 and N3D1were systematically mutated in complete gene plasmid. WT refers to the wild type VSR plasmids. (**c**) Graphical representation of GFP reversion assay. Mutated VSR plasmids were transfected in Sf21 sensor cell line for GFP reversion assay and FACS analysis was done after 72 h. The data represents Mean ± SD of FACS results. The *symbol indicates a statistically significant difference in terms of P value (P < 0. 001). Statistical significance was analysed using student’s t-test and Fisher’s Least Significant Difference (LSD) test using corresponding wild type (WT) plasmid as control. nsP2 mutated plasmid 3 (nsP2/M3 p-value < 0.0001) and nsP3 mutated plasmid 2 (nsP3/M2, p-value < 0.0001) were found highly significant (Alpha level of the test = 0.05). (**d**) EMSA using labeled GFPshRNA probe. CHIKV N2D2, N2M3, N3D1 and N3M2 were transfected in Sf21 cell line. GFPshRNA oligonucleotide was end-labelled with [γ32P] ATP and mixed with different concentrations of transfected Sf21 cell lysate. Lane 1, 10: Free shRNA probe; lane 2: nsP3 (30 μg), lane 3, 4, 6, 7: different concentrations of N3M2-Sf21 (20 μg, 30 μg) and N3D1-Sf21 (20 μg, 30 μg) respectively. Lane5, 8: N3M2(30 μg) and N3D1(30 μg) with 100 fold unlabelled GFPshRNA probe; lane 9: nsP1 Sf21 cell lysate (30 μg); lane 11, 12.14, 15: CHIKV N2D2 (30 μg, 20 μg) and N2M3 (20 μg, 30 μg)Sf21 lysate respectively; lane13: N2D2 Sf21 lysate (30 μg) with 100 fold unlabelled GFPshRNA probe. Salmon sperm DNA (2 μg) was used as non specific inhibitor in all binding reactions.

Having identified the conserved RNA binding motifs in nsP2 and nsP3, we proceeded to evaluate the impact these motifs have in RNAi suppressor activity in the VSRs. Substituting all or most of the amino acids in motifs with alanine through site directed mutagenesis, we generated mutants. Three mutants were generated in nsP2 with different permutations and combinations of substitutions, while two mutants were generated in nsP3. Schematic representation of mutant generation is depicted in Fig. 7a and b. GFP reversal in these mutants were evaluated using Sf21 RNAi sensor line and the results are shown in Fig. 7c. Further, we performed mobility shift assays to evaluate the binding capacities of the domains and the mutants generated using [γ32 P] labeled shRNA of GFP and running the bound complex in a 6% polyacrylamide gel (Fig. 7d). Complex formation due to dose dependent binding of shRNA to the mutants of nsP3 and nsP2 are evident (lane 3, 4, 14 and 15 respectively). In the same manner, binding efficiencies of the domains to shRNA are demonstrated in lane 6, 7, 11 and 12 respectively. The results clearly show the involvement of the RNA binding motifs in the binding to dsRNA and we conclude that these motifs play a direct role in RNAi suppressor activity. nsP1-Sf21 lysate in the presence of non-specific inhibitor (salmon sperm DNA) and GFPshRNA served as negative controls (lane 1, 9, 10).

## Discussion

Alphaviruses are maintained in nature by alteration between mosquitoes and human host. While their infections in mosquitoes are mainly asymptomatic and persistent, in vertebrate hosts, the infection is acute and self limiting^31^. Efficiency of virus replication in both vector and host depends not only on the expression of its own proteins but also on its ability to counteract upon host antiviral defence. Mosquito innate immune system poses a serious challenge to virus infection and activates different defence pathways including TOLL pathway, IMD (Immune deficiency) pathway, JAK STAT pathway and RNAi pathway^45^. RNAi pathway is a major antiviral defence mechanism employed by insects that results in degradation of viral RNA thereby curbing infection^1^,^2^,^3^,^4^. Viruses employ numerous strategies to evade host RNAi response, including mutations in or near the target to bring conformational changes rendering RISC inaccessible^46^ and VSRs which inhibit or compete with Dicer/other RNAi components, thus decreasing the amounts of viably loaded RISC complexes^7^,^46^,^47^.

Earlier reports have suggested that arboviruses may possess weak suppressors that have been yet undetected due to inappropriate assay conditions^4^,^37^. The speculations of absence of VSRs in arboviruses were eliminated when DENV proteins showed suppressor activity^48^. However, since alphaviruses do not cause as severe illness as the flavivirus, it was assumed that VSR activity against RNAi defence might be detrimental for the host, thus reducing virus transmission^36^,^49^. Using an in-house developed GFP reversion assay^16^,^26^, we report that CHIKV nsP2 and nsP3 are RNAi suppressors. Certain VSRs such as Geminivirus nuclear protein Ac2 have been previously shown to transactivate host genes^50^. We confirmed the specificity of the GFP reversion by checking nsP2 and nsP3 for non-specific promoter activity (Supplementary Fig. S1).

The non-structural proteins, nsP2 and nsP3 of CHIKV participate in several functions; nsP2 has been shown to have multiple functions including helicase, protease and NTPase activities^30^,^40^,^41^,^42^. nsP2 has been shown to interact the most with host factors than any other CHIKV protein, mainly due to its dual role as key component of viral replication complex and also as an important virulence factor inhibiting the host antiviral response^51^. In our study, nsP2 has shown stronger VSR activity in comparison to nsP3. Earlier reports have shown this protein exhibits anti-IFN activity^52^, emphasising on the overlapping nature of interferon and RNAi pathway. Domain mapping of this protein revealed the domain that possessed helicase activity showed maximum activity for RNAi suppression. Interestingly, helicases such as HeIf of Dictyostelium, B2 of Wuhan nodavirus and NS3 of dengue virus has been proven to exhibit VSR activity^15^,^48^,^53^. Of interest is the presence of a GW motif, conserved across all alphavirus species studied, that is known to be important for binding to dsRNA/siRNA and Argonaute loading^44^. The crystal structure of CHIKV nsP3 macrodomain reveals that this domain possesses RNA-binding activity^33^. This protein is also known to modulate pathogenicity in mice^54^. Our study has provided evidence of an additional role of the macrodomain and clearly elucidates it role in pathogenicity as a viral suppressor of RNAi. The existence of both nsP2 and nsP3 in both the nucleus as well in the cytoplasmic fraction^55^,^56^ may help in their interactions with the various components of the RISC complex.

Alphavirus species exhibit sequence conservation amongst several of their proteins. Motifs that are involved in direct interactions with both the vector as well as the host have been shown to be conserved even in the otherwise non-conserved regions^57^. Through our study we have established all RNA binding motifs of CHIKV nsP2 and nsP3 domains that possess VSR activity are conserved across alphavirus species. It is speculated that these proteins may participate in similar functions in the other alphavirus species as well.

Taken together, the current study has identified CHIKV proteins nsP2 and nsP3 to exhibit RNAi suppressor activity in the *in-vitro* system that was further demonstrated in its natural hosts, namely, an *Aedes* and mammalian cell line. The domains responsible for suppressor activity was further identified and their suppressor strength evaluated based on mutations introduced in the RNA binding motifs of these domains. Finally, these RNA binding motifs were found to be conserved across alphavirus species. It is to be noted however, the VSR activities of nsP2 and nsP3 exhibited by *in-vitro* assays could be strengthened further by a direct demonstration employing *in-vivo* infection system.

## Methods

### Cell Lines and Virus

Sf21 cell line was maintained in BD Baculogold complete TNM-FH insect media at 28 °C. Sf21 GFP and Sf21 GFPshRNA (sensor cell line) were maintained in the same media but with 50 μg/ml zeocin as an additional component. Aag2 cell line was maintained in Schneider’s media, supplemented with 10% FBS and 50 mg/ml of penicillin-streptomycin antibiotic, at 28 °C and 5% CO_2_. HEK293T cell line was maintained in Dulbecco’s Modified Eagle medium (DMEM) supplemented with 10% FBS, 2mM L-Glutamine, 50 mg/ml Pen Strep antibiotic at 37 °C and 5% CO_2_. Chikungunya virus (CHIKV) was isolated from viral stock made from patient sera sample IND-10-DEL9 infected in C6/36 and Vero cell line. The culture supernatant was used for CHIKV RNA isolation.

### Generation of Plasmid Constructs

All nine CHIKV genes were amplified with respective primers using Reverse Transcription PCR and cloned in pIB/V5-His TOPO vector (Invitrogen, USA), under OpIE2 promoter (a baculovirus early promoter) for transfections in Sf21 and Aag2 cell lines. The genes were also cloned in pCDNA 3.1+ vector (Invitrogen, USA) under CMV promoter using respective primers for transfections in HEK293T cell line. nsP2 and nsP3 were cloned in the plant binary vector pBI 121 under CaMV 35 S promoter for *Agrobacterium* mediated infiltration in *Nicotiana* leaves. Four domains of nsP2 and three domains of nsP3 were separately cloned in pIB/V5-His TOPO vector. Plasmids and primers used in this study are listed in Supplementary Table S1.

### RNAi Suppression Assay in Sf21 sensor cell line

To find the putative RNAi suppressors in CHIKV, a previously developed RNAi sensor cell line constitutively expressing GFPshRNA was used. Briefly, 0.2 million Sf21 GFPshRNA cells were seeded in a 24 well plate in complete media and transfected with 2 μg of CHIKV gene plasmids using CellfectinII reagent (Invitrogen) as per manufacturer’s protocol to transiently express the CHIKV proteins. Empty pIB/V5-His TOPO vector was used as negative control. Previously published dengue virus suppressor NS4B and flock house virus protein B2 (FHVB2) were used as positive controls. FACS analysis was done after 72 h for GFP expression in transfected cells. Sf21 cell line with and without GFP were used as FACS gating control. GFP specific dsRNA and siRNA mediated reversion assay was carried out similarly in Sf21 GFP stable cell line in 24 well plate. Briefly, 2 μg VSR plasmid was co-transfected with 1.5 μg GFP dsRNA plasmid and 2.5 pmole siRNA in separate wells. Scrambled siRNA was used as GFP silencing negative control. FACS analysis was done similarly after 72 h.

### RNAi Suppression Assay in natural host cell lines

Aag2 (*Aedes aegypti*) cells were transfected using Attractene reagent (Qiagen). Briefly, 0.2 million Aag2 cells were co-transfected with GFPshRNA plasmid and CHIKV-pIB/V5-His TOPO plasmid. GFP levels were analyzed with FACS after 72 h. GFP reversion assay in human host was done using HEK293T cells. Each CHIKV-pCDNA3.1+ plasmid was co-transfected with GFPshRNA plasmid using Jet PRIME reagent (Polyplus Transfection) in jet PRIME buffer, followed by FACS analysis after 72 h.

### FACS Analysis of various cell lines

GFP levels in different cell lines were analysed by Fluorescence Activated Cell Sorting (FACS) with FACS calibur (BD Biosciences). Media from cells was removed and the cells were washed and resuspended in 500 μl of FACS grade PBS. Respective cell lines without GFP were used specifically for gating the region of negative control. Cell Quest software (Becton-Dickinson, Franklin Lakes, NJ, USA) was used to analyse the percentage of GFP reversion in 20,000 cells against FL1-H.Percentage GFP reversion data was represented as Mean ± SD. The experiments were repeated at least four times in triplicates. Statistical analysis of experimental data was conducted using GraphPad Prism (version 6). Two tailed student’s t test and Fisher’s Least Significant Difference (LSD) test were performed to check significance of the data and p-values < 0.001 were considered significant.

### Validation of RNAi suppression in *Nicotiana* leaves

nsP2 and nsP3 genes were separately cloned in pBI 121 vector for transformation in *Agrobacterium*. The secondary culture was incubated in fresh YEM and acetosyringone for 1 h, followed by infiltration in transgenic *Nicotiana* leaves which has GFPshRNA stably integrated. The culture was infiltrated on dorsal side of the leaves. The leaves were analysed in UV transilluminator 3, 5, 7 and 10 days post infiltration (dpi). A previously known suppressor FHVB2 was used as positive control and mutated FHVB2 was used as negative control.

### Detection of CHIKV proteins, VSR mutants and domains in Sf21 cells

Sf21 cell line was transfected with all 9 genes of CHIKV cloned in pIB vector, nsP2/M3 pIB, nsP3/M2 pIB and VSR domains cloned in pIB plasmids separately. The cells were harvested after 72 h and lysed in Lysis buffer (50 mM Tris HCl and 300 mM NaCl) containing Protease Inhibitor cocktail (Roche). Whole cell lysate concentration was determined using Bradford protein assay (Bio-Rad laboratories). Protein extracts (60 μg) were subjected to 12% reducing PAGE and Western Blotting was done using anti-His (mice) primary antibody (Sigma), followed by AP conjugated (mice) secondary antibody.

### Western Blotting to detect changes in GFP expression levels

Sf21sensor cell line was transfected with nsP2 pIB and nsP3 pIB plasmids separately and cells were harvested after 72 h. 60 μg total cell lysate was run on 12% SDS PAGE and Western Blotting was done using anti-GFP primary antibody (Santa Cruz) and AP conjugated secondary antibody. Levels of GAPDH proteins were used as housekeeping control.

### Electrophoretic mobility shift assay

GFPshRNA binding ability of of different proteins was evaluated using Electrophoretic mobility shift assay (EMSA). Sf21 cells transfected with nsP2, nsP3, mutants and domains plasmids were analysed along with untransfected Sf21 cell line as negative control. GFPshRNA probe was synthesised from Sigma as a self complementary 49 bp DNA oligonucleotide with a hairpin loop. Binding reaction was setup with different concentrations of cell lysate, 1X Binding buffer (30 mM HEPES and 100 mM NaCl), [γ^−32^P] ATP labelled shRNA probe (20,000 cpm per reaction) and 2 μg of salmon sperm DNA (Thermo Scientific). Unlabelled shRNA was added as cold probe to check specificity of the binding. The complex was resolved on 6% polyacrylamide non-denaturing gel (pre-run for 1 h at 4 °C) in 1X TBE buffer at 100 V at 4 °C. Gels were dried and exposed for auto-radiography. The scanning was performed using Typhoon 9210 Variable mode imager (Amersham Biosciences).

### Northern Blotting

*Nicotiana* leaves infiltrated with VSR plasmids were harvested and total RNA was isolated using GITC method. For mRNA Northern. 20 μg total RNA was run on 1% agarose gel. RNA was transferred onto Hybond-N^+^ membrane (GE Healthcare) overnight by capillary transfer. RNA was immobilised on the membrane by UV cross linking at 1,200 × 100 μJ. Northern blotting for mRNA was performed using [γ^−32^P] ATP labelled shRNA probe.

For small RNA northern. RNA was isolated using GITC method followed by small RNA enrichment. 50 μg RNA was run on 15% PAGE containing 8 M Urea. RNA was transferred onto a Hybond-N^+^ membrane (GE Healthcare) for 60 min at 15 V and immobilised by UV cross-linking at 1,200 × 100 μJ. GFP probe was prepared by DIG labelling kit (Roche, USA). Northern hybridisation and development was performed according to instruction manual. Small RNA marker (NEB) lane was cut from the blot and radioactively labelled with [γ^−32^P] ATP separately.

### Domain mapping of the CHIKV RNAi suppressors in sensor cell line

Sf21 sensor cell line was transfected with 2 μg of each domain plasmid using Cellfectin II reagent, followed by FACS analysis after 72 h. Complete gene plasmid and FHVB2 were used as positive controls and empty vector was used as negative control.

### Alignment of RNA binding motifs of alphaviruses

nsP2 and nsP3 sequences of 14 other alphaviruses were fetched from NCBI and aligned with CHIKV New Delhi strain IND-10-DEL9 using MEGA5 software. The RNA binding motifs were predicted using BindN software and checked for conservation amongst alphaviruses.

### Site Directed Mutagenesis in nsP2 and nsP3 pIB/V5-His TOPO plasmids

nsP2 and nsP3-pIB clones were mutated using QuickChange multi site directed mutagenesis kit (Agilent Technologies) for mutating different sites in the same plasmid simultaneously. Briefly, 50ng plasmid was mutated using different primers binding to the same DNA strand with PCR, followed by *DpnI* digestion and transformation. The mutations were confirmed by sequencing from Macrogen. GFP reversion was assayed in Sf21 sensor cell line. Wild type VSR plasmids were used as positive controls.

## Additional Information

**How to cite this article**: Mathur, K. *et al*. Analysis of chikungunya virus proteins reveals that non-structural proteins nsP2 and nsP3 exhibit RNA interference (RNAi) suppressor activity. *Sci. Rep.* **6**, 38065; doi: 10.1038/srep38065 (2016).

**Publisher's note:** Springer Nature remains neutral with regard to jurisdictional claims in published maps and institutional affiliations.

## Supplementary Material

Supplementary Information
