# Supplementary material for: Analysis of chikungunya virus proteins reveals that non-structural proteins nsP2 and nsP3 exhibit RNA interference (RNAi) suppressor activity
Source: Sci Rep. 2016 Nov 30;6:38065. doi: 10.1038/srep38065 (PMC5128919; doi:10.1038/srep38065)
Supplement: Supplementary Information [file srep38065-s1.pdf]

Analysis of chikungunya virus proteins reveals that non-structural proteins nsP2 and nsP3 exhibit RNA interference (RNAi) suppressor activity

Kalika Mathur[1], Abhishek Anand[2], Sunil Kumar Dubey[1], Neeti Sanan-Mishra[2], Raj K Bhatnagar[1*], Sujatha Sunil[1*]

Supporting information

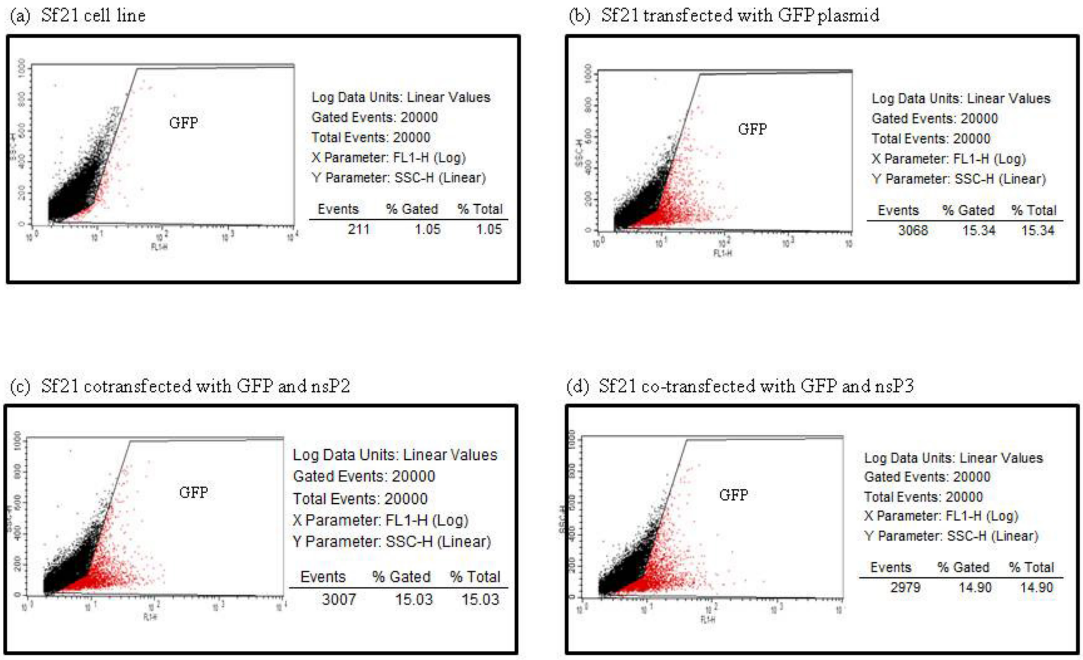

Supplementary Figure S1. Trans-activation assay of CHIKV VSRs. Sf21 cell line was co-transfected with GFP plasmid and VSR plasmids. FACS analysis was done after 72h.

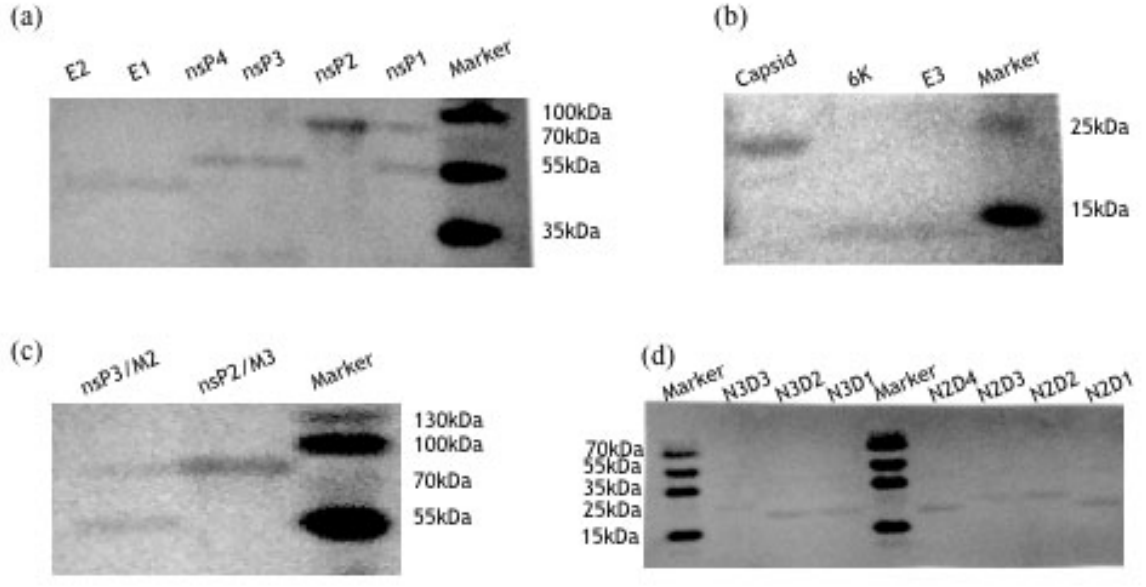

Supplementary Figure S2. Western blotting of CHIKV plasmid constructs. Sf21 cell line was transfected with CHIKV plasmids. Cells were harvested after 72h and western blotting was done using anti-His primary antibody and AP conjugated secondary antibody.

Supplementary Table 1. Primers used in the study

S.No.	Plasmid	Primer Sequence
	**pIB/V5-His TOPO (Transfection in Sf21 & Aag2)**	
1	nsP1	FP: GTAATGGATCCTGTGTACGTGG<br>RP: TGCACCCGCTCTGTCCT
2	nsP2	FP: GTAATGGGAATAATAGAGACTCCGAGA<br>RP: TCCTGCTCGGGTGGCCTG
3	nsP3	FP: GTAATGGGATGTGCACCGTCGTACCGG<br>RP: TTACTCGTCGTCCGTGTCTG
4	nsP4	FP: GTAATGGGACGACTAGACAGGGCAGGTG<br>RP: AGGACCGCCGTACAAAGTTA
5	E1	FP: GTAATGGCGTACGAACACGTAACAG<br>RP: TTAGTGCCTGCTGAACGACAC
6	E2	FP:GTAATGGGAAGCACCAAGGACAACTTCAAT<br>RP: TTTAGCTGTTCTGATGCAGC
7	E3	FP: GTAATGGGATGGAGTCTTGCCATCCCAGT<br>RP: GCGTCGCTGGCGGTGG
8	Capsid	FP: GTAATGGAGTTCATCCCAACCC<br>RP: CTCTTCGGCTCCCTCAG

9	6K	FP: GTAATGGCGGCCACATACCAAGAG<br>RP: GCTCACAGTGTGGGCAC
10	N2D1	FP: GTAATGGGAATAATAGAGACTCCGAGA<br>RP: GACTGCAATTTTGTATGGGCAG
11	N2D2	FP: ATCGGATCCGTAATGGGAATAGGAGTCTTCG<br>RP: TGTCTTCCATACCAGTTTACCTTC
12	N2D3	FP: ATCGGATCCGTAATGGGACTCTCCGGTGACC<br>RP: CTTGTTAACCAGCCATTCCATTC
13	N2D4	FP: ATCGGATCCGTAATGGGAATAAACGGCCACCA<br>RP: TCCTGCTCGGGTGGCCTG
14	N3D1	FP: GTAATGGGATGTGCACCGTCGTACCGG<br>RP: TTGGGTCCGCATCTGTAT
15	N3D2	FP: ATCGGATCCGTAATGGTGGAGCTGCTGG<br>RP: GTATTCCCTTGGACTTACGCG
16	N3D3	FP: ATCGGATCCGTAATGGGAAGACCTTCCCAGGAGT<br>RP: TTACTCGTCGTCCGTGTCTG
	**pcDNA 3.1(+) (Transfection in HEK293T)**	
17	nsP1	FP:ATCAAGCTTGTAATGGATCCTGTGTACGTGG<br>RP: ATCGAATTCATTTGCACCCGCTCTGTCCT
18	nsP2	FP:ATCGGATCCGTAATGGGAATAATAGAGACTCCGAGA<br>RP: ATCCTCGAGATTTCCTGCTCGGGTGGC
19	nsP3	FP: ATCGGATCCGTAATGGGATGTGCACCGTCGTA<br>RP: ATCGAATTCATTTAACTCGTCGTCCGTGTCTG
20	nsP4	FP: ATCGGATCCGTAATGGGACGACTAGACAGGG<br>RP: ATCGAATTCATTAGGACCGCCGTACAAAGTTA
21	E1	FP: ATCGGATCCGTAATGGCGTACGAACACGTAACAG<br>RP: ATCGAATTCATTGTGCCTGCTGAACGACAC
22	E2	FP: ATCGGATCCGTAATGGGAAGCACCAAGGACA<br>RP: ATCGAATTCATTTTTAGCTGTTCTGATGCAGC
23	E3	FP:ATCGGATCCGTAATGGGATGGAGTCTTGCCATCCCAGT<br>RP: ATCGAATTCATTGCGTCGCTGGCGGTG
24	Capsid	FP: ATCGGATCCGTAATGGAGTTCATCCCAACCCA<br>RP: ATCGAATTCATTCTCTTCGGCTCCCTCAG
25	6K	FP: ATCGGATCCGTAATGGCGGCCACATACCAA<br>RP: ATCGAATTCATTGCTCACAGTGTGGGCAC
26	**shRNA oligonucleotide**	GGT TAT GTA CAG GAA CGC ATT CAA GAG ATG CGT TCC TGT ACA TAA CCT T
